# The guiding role of gastric filling ultrasonography in the diagnosis of stromal tumors and schwannomas—a retrospective study

**DOI:** 10.3389/fonc.2026.1680846

**Published:** 2026-03-30

**Authors:** Yan Wang, Xuemei Qi, Lingling Deng, Cuihong Liu, Shaoling Liu

**Affiliations:** Department of Ultrasound, Shandong Provincial Hospital Affiliated to Shandong First Medical University, Jinan, China

**Keywords:** gastric filling ultrasonography, gastric schwannoma, gastric stromal tumor, risk stratification, ultrasonographic characteristics

## Abstract

**Objective:**

To differentiate the ultrasonographic characteristics of gastric stromal tumors (GSTs) and gastric schwannomas (GSs) identified via gastric filling ultrasonography and investigate the relationship between National Institutes of Health (NIH) risk stratification and the ultrasonographic imaging characteristics of GSTs.

**Methods:**

A retrospective analysis was performed on the clinical and ultrasonographic imaging data of 77 patients with GSTs and 16 with GSs who were evaluated between 2020 and 2025.

**Results:**

Statistically significant differences were observed in ultrasonographic findings, including tumor diameter, internal echo, morphology, and boundaries, between the GST and GS groups. Moreover, significant differences in the ultrasonographic features of GSTs were found across different NIH risk stratifications. Intermediate- or high-risk GSTs were closely associated with larger tumor diameters, more heterogeneous internal echoes, irregular shapes, indistinct boundaries, and abundant intratumoral blood flow. A scoring system was established based on significant ultrasonographic features derived from ROC curve analysis.

**Conclusions:**

Based on our analysis, gastric filling ultrasonography can be utilized to differentiate GSTs from GSs and predict the risk stratification of GSTs through an established ultrasound image scoring system. The clinical application of gastric filling ultrasonography may aid in the preoperative diagnosis of GSTs and the development of individualized treatment plans.

## 1 Introduction

Gastric stromal tumors (GSTs) and gastric schwannomas (GSs) are mesenchymal tumors classified as gastric submucosal lesions, primarily arising from the muscularis propria. GSs account for only 0.2% of all gastric tumors; they tend to grow slowly, rarely recur or metastasize following surgical intervention, and generally have a favorable prognosis. In contrast, GSTs are the most common mesenchymal tumors of the stomach and possess malignant potential. Surgical resection is required for patients preoperatively assessed as high risk, and targeted drug therapy is often necessary before or after surgery. Given the differences in treatment strategies and prognostic outcomes between GSTs and GSs, it is crucial to establish a diagnosis prior to initiating treatment. However, most GSTs and GSs are challenging to diagnose using routine gastroscopy, as both appear as submucosal protrusions.

Gastric filling ultrasonography involves a comprehensive examination of the stomach’s interior, including the gastric wall layers and perigastric structures, following the ingestion of an oral contrast agent. Through direct visualization, this technique enables the precise identification of the origin and characteristics of gastric lesions, particularly those located in the submucosal layer. Consequently, the diagnostic accuracy of gastric filling ultrasonography is significantly higher than that of routine gastroscopy (1, 2). This study was conducted to analyze the sonographic characteristics of GSTs and GSs and to explore the role of gastric filling ultrasonography in differentiating between these two entities.

## 2 Materials and methods

### 2.1 Data collection

Clinical data and ultrasonographic imaging information were retrospectively collected from patients with gastric stromal tumors (GSTs) or gastric schwannomas (GSs) who were pathologically confirmed between June 2020 and June 2025 at Shandong Provincial Hospital. The collected data included patient demographics and tumor characteristics such as size, boundary, and internal echogenicity. This study was approved by the Biomedical Research Ethics Committee of Shandong Provincial Hospital (Approval No. SWYX: NO.2025-447). As the data were collected retrospectively and did not involve patient privacy, the committee granted a waiver of informed consent. Patients were included if they underwent gastric filling ultrasonography after administration of a gastrointestinal ultrasound contrast agent and were subsequently diagnosed with either a GST or a GS by pathological examination. The exclusion criteria were as follows: (1) patients diagnosed with a GST or a GS who did not undergo gastric filling ultrasonography or who received water-filled ultrasonography instead; (2) patients with incomplete clinical or imaging records.

### 2.2 Method of examination

Ultrasonography was performed using a GE LOGIQ Fortis Color Doppler Diagnostic System equipped with a low-frequency probe (3.0–5.0 MHz) and a linear array probe (3.0–12.0 MHz). After fasting for more than 8 hours, patients were orally administered 500–600 mL of a dissolved gastrointestinal ultrasound contrast agent (Huzhou East Asia Pharmaceutical Co., Ltd.) to fill the gastric cavity prior to the examination. The contrast agent was prepared at a ratio of 1:9 (contrast medium to water). Gastric filling ultrasonography was performed with the patient in various positions, including sitting, supine, and right lateral decubitus, to observe the abdominal segment of the esophagus, cardia, gastric fundus, body, antrum, pylorus, and duodenum. When a lesion was detected, its anatomical location, originating layer, size, growth pattern, morphology, margins, internal echogenicity (including ulcers, cystic degeneration, and calcifications), and the status of perigastric lymph nodes were assessed and recorded. A high-frequency probe was subsequently used to observe lesion details, and color flow Doppler imaging (CDFI) was employed to evaluate blood flow distribution. Blood flow was graded according to the Adler semiquantitative method (3) as follows: Grade 0 indicated no significant intratumoral blood flow; Grade I indicated 1–2 punctate blood flow signals, representing low blood flow; Grade II indicated punctate or a single strip-like blood flow signal, representing moderate blood flow; and Grade III indicated multiple thick blood flow signals, indicating abundant vascularity. All imaging data were archived in the hospital’s Picture Archiving and Communication System (PACS).

### 2.3 Statistical analysis

Statistical analysis was conducted using SPSS 21.0 software. ROC curve was made by Graphpad Prism 9.0. Continuous variables were analyzed by the Mann-Whitney U test subsequent to Shapiro-Wilk normality testing, whereas categorical variables were analyzed by chi-square analysis, Fisher’s exact test and Kruskal-Wallis test. A *p* value < 0.05 was considered statistically significant.

## 3 Results

### 3.1 Clinical data analysis

This study collected records from 107 patients with GSTs and GSs and excluded 14 cases on the basis of the exclusion criteria. Among the remaining 93 cases, patients of GSTs were 77cases, including 32 males and 45 females, and the average age was 62.4 ± 12.6 years. According to the National Institutes of Health (NIH) consensus classification system, the GST risk stratification was divided into four levels: very low, low, intermediate and high risk. Among the 77 patients with GSTs, 43(55.8%) cases were in the very low-risk and low-risk groups, 22 (28.6%) cases were in the intermediate-risk group, 12 cases were in the high-risk group. There were 16 cases with GS, including 6 males and 10 females, and the average age was 53.2 ± 10.7 years. There was no significant difference in the sex ratio between the GST and GS groups (*p* = 0.527>0.05), but significant differences were observed in age by the Mann–Whitney U test (*p* = 0.016<0.05) (**Table 1**).

**Table 1:** Clinical data analysis.

Model	GST (n=77)	GS (n=16)	*p* value
Sex, n (%)			0.527
Male	32 (41.6%)	6 (37.5%)	
Female	45 (58.4%)	10 (62.5%)	
Age (years)	62.4 ± 12.6	53.2 ± 10.7	*0.016*
Risk stratification (GST only), n (%)			
Very low or low	43 (55.8%)	—	
Intermediate	22 (28.6%)	—	
High	12 (15.6%)	—	

### 3.2 Ultrasonographic image characteristics

**Figure 1:**
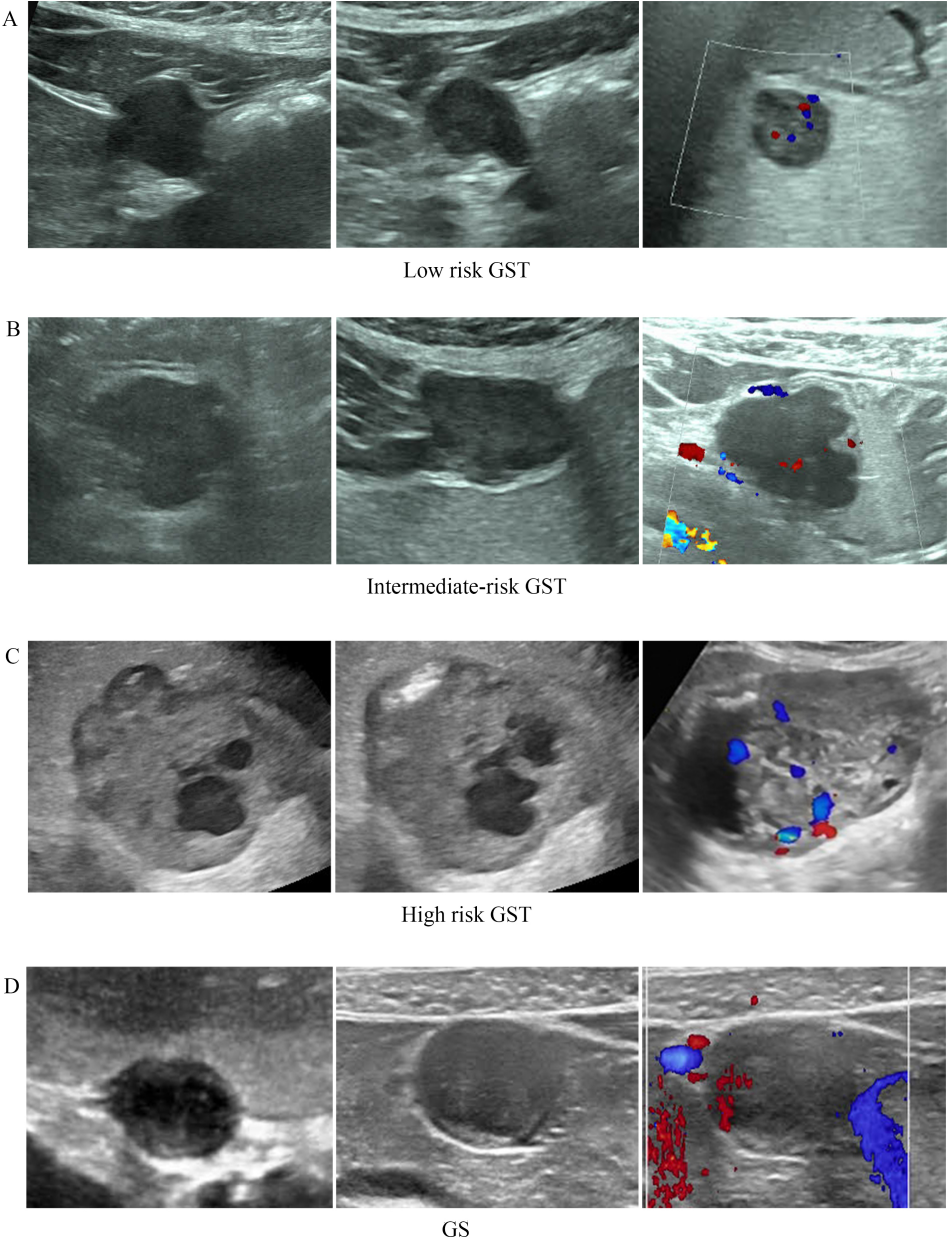
Sonography of different group of GST and GS. **(A)** The diameter of GST was 2.9cm with regular shape, clear boundary and homogeneous internal echo, tumor was extended into the muscularis propria. The CDFI was grade II; **(B)** The diameter of GST was 6.2cm with lobular shape, blurred boundary and heterogeneous internal echo, tumor was extended into the muscularis propria. The CDFI was grade II; **(C)** The diameter of GST was 12cm with irregular shape, clear boundary and heterogeneous internal echo (cystic-solid). The CDFI was grade III; **(D)** The diameter of GS was 2.5cm with regular shape, clear boundary and homogeneous internal echo. The CDFI was grade I.

This study analyzed the ultrasonographic image characteristics of GSTs and GSs, and the findings revealed similarities in their occurrence layer and blood flow status (*p=*>0.05). However, there were significant differences in tumor size, morphology, boundaries, internal echoes and primary sites (*p* < 0.05) (**Table 2**). The average diameter of the GSTs was 6.9 cm, and whereas that of the GSs was 3.9 cm (*p* < 0.001). The differences in internal echoes between the two tumors was statistically significant (*p* = 0.043<0.05). GSTs were mostly heterogeneously hypoechoic (41.6% of cases showed homogeneous echoes, and 58.4% showed heterogeneous echoes), with fluid-filled areas and hyperechoic spots. However, the internal echoes of the GSs were mostly homogeneous, with only a few occurrences of hyperechoic spots and liquid components (68.75% of cases showed homogeneous echoes and 31.25% showed heterogeneous echoes). Differences in morphology were noted (*p=*0.011 <0.05), and the GSTs exhibited more irregular shapes than the GSs did. Among the GSTs, 62.3% had an irregular and lobulated morphology, while this proportion was only 25% in GSs. A significant difference was also observed in the boundary between the two (*p* = 0.002 < 0.05): nearly all GSs had clear boundaries, whereas unclear boundaries were present in some GSTs (37.7% of GSTs had unclear boundaries, and all GSs had clear boundaries). The differences in ultrasonographic features between GSTs and GSs are demonstrated in **Figure 1**.

**Table 2:** Comparison of ultrasonic imaging characteristics between GSTs and GSs.

Model	GST (n=77)	GS (n=16)	*p* value
Average diameter (cm)	6.9 ± 3.8	3.9 ± 2.0	<0.001
Morphology, n (%)			0.011
Regular	29 (37.7%)	12 (75.0%)	
Irregular	48 (62.3%)	4 (25.0%)	
Internal echo, n (%)			0.043
Homogeneous	32 (41.6%)	11 (68.8%)	
Heterogeneous	45 (58.4%)	5 (31.2%)	
Boundary, n (%)			0.002
Clear	48 (62.3%)	16 (100.0%)	
Unclear	29 (37.7%)	0 (0.0%)	
Layer	Muscularis propria (all)	Muscularis propria (all)	—
Blood flow (Adler), n (%)			0.320
Grade 0	21 (27.3%)	6 (37.5%)	
Grade I	24 (31.2%)	5 (31.2%)	
Grade II	21 (27.3%)	4 (25.0%)	
Grade III	11 (14.3%)	1 (6.2%)	
Location, n (%)			<0.001
Gastric fundus	19 (24.7%)	0 (0.0%)	
Gastric antrum	22^a^ (28.6%)	6^b^ (37.5%)	
Greater curvature	13 (16.9%)	3 (18.8%)	
Lesser curvature	23 (29.9%)	1 (6.2%)	
Anterior wall of gastric body	0 (0.0%)	2 (12.5%)	
Posterior wall of gastric body	0 (0.0%)	4 (25.0%)	

a Three cases at the junction of the gastric antrum and gastric body.

b Two cases at the junction of the gastric antrum and gastric body.

In addition, there were significant differences in the primary site between the GSTs and the GSs (*p* < 0.001). GST mainly occurs in the lesser curvature of the stomach, gastric antrum and gastric fundus. Among GST cases, 29.9% occurred on the lesser curvature of the stomach, 16.9% occurred on the greater curvature, 28.6% occurred in the gastric antrum, and 24.7% occurred in the gastric fundus; no GST was identified in the anterior or posterior wall of the gastric body in this study. Among the GSs, 37.5% occurred in the gastric antrum, 12.5% occurred in the anterior wall of the gastric body, 25% occurred in the posterior wall of the gastric body, 6.25% occurred on the lesser curvature side of the gastric body, and no lesions were identified in the gastric fundus. These findings indicate that GSs are concentrated mainly in the anterior and posterior walls of the gastric body and antrum and rarely occur in the upper portion of the stomach. However, there was no significant specificity in the localization of GSTs, except for their relatively infrequent occurrence on the anterior wall, posterior wall, and greater curvature of the gastric body. GSTs and GSs did not differ significantly in blood flow status (*p=*0.32>0.05); both tumor types demonstrated relatively low blood flow. The GSTs with a relatively large volume exhibited abundant blood flow, whereas there was no significant correlation between the blood flow distribution within the GSs and their volume.

### 3.3 Ultrasound image feature analysis based on GST risk stratification

The risk stratification of GSTs varies significantly with tumor size, internal echoes, morphology, boundaries, and blood flow distribution but not with tumor location. The ultrasound imaging features of GSTs with different risk stratifications are presented in **Figures 1A–C**, and the statistical results are listed in **Table 3**. In this study, a larger tumor size generally was correlated with a higher risk stratification, indicating a positive correlation between the two factors (*p* = 0.001<0.05). The average diameter of the tumors in the very low- and low-risk groups was 4.6 cm, that in the intermediate-risk group was 8.4 cm, and that in the high-risk group was 12.5 cm. The heterogeneity of internal echoes was positively correlated with risk stratification (*p* = 0.001<0.05). From very low/low to high risk, the proportions of internal echo homogeneity were 37.2%, 77.3%, and 100%, respectively. Moreover, risk stratification was closely related to tumor morphology and boundaries. High-risk GSTs often have an irregularly shaped morphology and blurred margins. Statistical analysis revealed that 39.5% of tumors in the low-risk group had an irregular morphology and 20.9% had an unclear boundary; 86.4% of tumors in the intermediate -risk group presented with an irregular morphology and 54.5% with unclear boundaries; while 100% of tumors in the high-risk group exhibited an irregular morphology and 66.7% had unclear boundaries. Similarly, the blood flow distribution of tumors was also related to risk stratification. We observed a strange phenomenon: in the low- and intermediate-risk groups, the degree of blood flow abundance was positively correlated with tumor risk stratification, but in the high-risk group, blood flow was decreased. This reduced blood flow may be related to rapid tumor growth, excessive tumor volume, or tumor necrosis in the central part. Additionally, there was no significant difference in the primary anatomical site among the GSTs with different risk stratifications (*p* = 0.317 > 0.05).

**Table 3:** Analysis of the correlation between ultrasonic imaging characteristics and risk stratification of GST.

Model	Low (n=43)	Intermediate (n=22)	High (n=12)	*p* value
Average diameter (cm)	4.6 ± 1.8	8.4 ± 3.1	12.5 ± 3.1	0.001
Internal echoes, n (%)				0.001
Homogeneous	27 (62.8%)	5 (22.7%)	0 (0.0%)	
Heterogeneous	16 (37.2%)	17 (77.3%)	12 (100.0%)	
Morphology, n (%)				0.001
Regular	26 (60.5%)	3 (13.6%)	0 (0.0%)	
Irregular	17 (39.5%)	19 (86.4%)	12 (100.0%)	
Boundary, n (%)				0.002
Clear	34 (79.1%)	10 (45.5%)	4 (33.3%)	
Unclear	9 (20.9%)	12 (54.5%)	8 (66.7%)	
Blood flow (Adler), n (%)				0.002
Grade 0	19 (44.2%)	2 (9.1%)	0 (0.0%)	
Grade I	14 (32.6%)	7 (31.8%)	3 (25.0%)	
Grade II	8 (18.6%)	7 (31.8%)	6 (50.0%)	
Grade III	2 (4.7%)	6 (27.3%)	3 (25.0%)	
Location (GST only), n (%)				0.317
Gastric fundus	12 (27.9%)	5 (22.7%)	2 (16.7%)	
Gastric antrum	14 (32.6%)	6 (27.3%)	2 (16.7%)	
Greater curvature	5 (11.6%)	3 (13.6%)	5 (41.7%)	
Lesser curvature	12 (27.9%)	8 (36.4%)	3 (25.0%)	

### 3.4 Drafting ultrasound scoring system of GST risk stratification based on ROC curve

**Figure 2:**
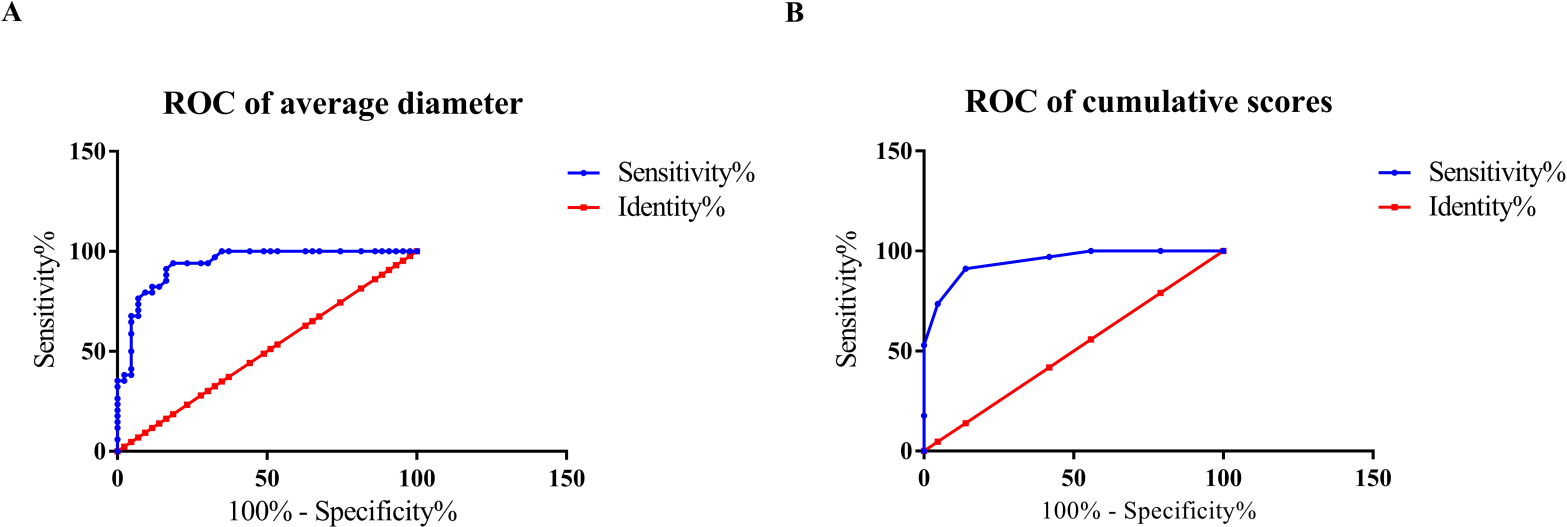
ROC curve of average diameter and cumulative scores. **(A)** ROC curve of average diameter for different GST risk stratification; **(B)** ROC curve of cumulative scores for different GST risk stratification.

We attempted to establish the ultrasound scoring system based on the cumulative scores of ultrasound image features of different GST risk groups and predict the GST risk stratification through ROC curves with “intermediate/high risk” defined as the positive outcome and “low risk” as the negative outcome. The ultrasound imaging features of tumors were comprehensively evaluated in terms of diameter, morphology, internal echo, boundary, blood flow distribution, and primary location (**Table 4**). One point was added for each feature that met the corresponding criterion, and the total accumulated points constituted the final score. Firstly, ROC curve analyzed the diagnostic performance of average tumor diameter in distinguishing very low/low-risk and intermediate/high-risk GSTs, its cut-off value was 5.45cm and AUC was 0.935 (sensitivity 94.12%, specificity 81.4%), as shown in **Figure 2A** and **Table 5**. Then, the cumulative score was calculated by adding the score of morphology, boundary, internal echo, average diameter, and blood flow status which were shown in **Table 4** below. Secondly, we adopted the cumulative scores to drawing ROC curve to determine the cut-off value between very low/low groups and intermediate-high risk groups, the cut-off value of cumulative scores was 3.5 with AUC = 0.959 (sensitivity 91.18%, specificity 86.05%) (**Figure 2B**; **Table 5**). Therefore, when the average diameter of the tumor was greater than 5.45cm or the cumulative score was greater than 4, the GST risk stratification can be classified as intermediate or high.

**Table 4:** The score rule of GST risk ultrasound image features.

Model	Score
Average diameter > 5.45cm	1
Heterogeneous internal echoes	1
Internal echoes with strong echo spots	1
Unclear boundary	1
Irregular morphology	1
Blood flow status	–
Grade 0	0
Grade I	1
Grade II or grade III	2

**Table 5:** ROC summary (GST: low vs intermediate/high).

Model	AUC	95% CI (bootstrap)	Cut-off	Sensitivity	Specificity
Diameter for low vs intermediate/high	0.935	0.873–0.980	5.45 cm	94.12%	81.40%
Cumulative score for low vs intermediate/high	0.959	0.918–0.988	3.5 (≥4)	91.18%	86.05%

### 3.5 Sensitivity analysis

Given the unbalanced sample sizes between the two groups in this study, to address the unstable estimation of conventional Logistic regression caused by the small sample size of GS and complete separation, Firth’s penalized Logistic regression was additionally adopted to estimate the odds ratio (OR) with 95% confidence interval (95%CI) as shown in **Table 6**. In the univariate model, for each 1 cm increase in tumor diameter, the OR for a diagnosis of GST was 1.53 (95%CI: 1.16–2.22, P<0.001), indicating that larger tumors were more likely to be GST. Unclear boundary was significantly associated with GST, with an OR of 20.07 (95%CI: 2.54–2594.65, *p* = 0.001176). In the multivariate model incorporating tumor diameter, boundary, morphology and internal echogenicity, unclear boundary remained an independent associated factor for GST (OR = 13.33, 95%CI: 1.61–1739.39, *p* = 0.0106). Tumor diameter was marginally significant in the multivariate model (OR = 1.52, 95%CI: 1.00–2.65, *p* = 0.0516), suggesting that the effect of tumor diameter might be partially confounded by other imaging features. Neither tumor morphology nor internal echogenicity reached statistical significance in the multivariate model (*p*>0.05). Subsequently, we performed a sensitivity analysis for sample imbalance. After enforcing sample balance between the two groups (16 vs. 16) and conducting 2000 repeated samplings, tumor diameter and boundary remained statistically significant in the vast majority of repetitions, indicating that these two variables had strong robustness. In contrast, the frequency of statistical significance for morphology and internal echogenicity decreased markedly after sample balancing (**Table 7**).

**Table 6:** Firth penalized logistic regression for discriminating GST vs GS.

Model	Predictor	OR (95% CI)	*p* value
Univariable Firth	Diameter (per 1 cm)	1.53 (1.16–2.22)	<0.001
	Boundary (unclear vs clear)	20.07 (2.54–2594.65)	0.001
Multivariable Firth	Diameter (per 1 cm)	1.52 (1.00–2.65)	0.052
	Boundary (unclear vs clear)	13.33 (1.61–1739.39)	0.011
	Morphology (irregular vs regular)	1.21 (0.26–6.00)	0.812
	Echo (heterogeneous vs homogeneous)	0.69 (0.14–3.17)	0.639

**Table 7:** Sensitivity analysis by repeated down-sampling (balanced 16 vs 16).

Feature	Proportion of P < 0.05 (2000 repeats)
Diameter	0.913
Boundary	0.795
Morphology	0.367
Internal echo	0.084

## 4 Discussion

Because conventional gastroscopy can only observe the luminal surface of the gastric wall, it is difficult to distinguish whether protuberant gastric lesions are caused by gastric compression by extrinsic space-occupying lesions or by gastric submucosal tumors. Therefore, conventional gastroscopy has certain limitations in the diagnosis of gastric submucosal tumors. Gastric filling ultrasonography can serve as a complement to gastroscopy to help differentiate gastric protuberant lesions. Furthermore, gastric filling ultrasonography can be used to locate the tumor layers precisely and identify tumor infiltration into surrounding tissues, lymph node involvement, and distant metastases (3).

It is known that GSTs originate from Cajal cells, which exist in connective tissues of the muscular layer of the gastrointestinal tract and generate electrical signals; these cells are pacemaker cells for gastrointestinal motility and are involved in the reception and transmission of neural signals (4). The GSs originate from Schwann cells in the Auerbach nerve plexus in the intermuscular layer of the gastric wall. Schwann cells are glial cells in the peripheral nervous system that participate in the formation of nerve fibers. Schwann cells can secrete neurotrophic factors, assisting in the repair of damaged neurons and promoting axonal regeneration (5). The GS is a benign tumor, while the malignant potential of a GST varies according to different risk stratifications. Consequently, the treatment and prognosis of these two tumors are very different. Small-volume GSs typically do not require any specific treatment, whereas larger-volume GSs that cause compressive symptoms in surrounding tissues and organs can be treated with surgical resection with a low risk of recurrence and a favorable prognosis (6). The treatment and prognosis of low-risk GSTs are analogous to those of GSs, while intermediate-risk GSTs carry certain risks, are primarily treated by surgical resection without subsequent treatment, necessitate close observation, and have a usually favorable prognosis. However, a high-risk GST is essentially equivalent to a malignant tumor, with a high risk of recurrence and distant metastasis and a poor prognosis. Following surgical resection of a high-risk GST, targeted immunotherapy can be administered according to the type of tumor mutation, and close observation is needed (7, 8).

This study revealed that the characteristics of the ultrasonographic images of GSTs and GSs obviously differed in morphology, the presence of internal echoes, tumor margins and anatomical location. Morphologically, the majority of GSs were regular and oval shaped, whereas the GSTs predominantly presented with a shallow lobulated appearance. This result differed from the reported shapes of GSTs and GSs of other studies (2). This discrepancy may be because a smaller GST tends to have a regular shape, being round or oval, whereas a larger GST tends to have an irregular shape. In this study, the majority of the GSTs were relatively large, with average diameters exceeding 3 cm. Moreover, small hypoechoic nodules found via ultrasonography are often not verified by further pathological examination; thus, these nodules were excluded from this study, which has led to a deviation between the study results and the theoretical understanding of GST morphology. Additionally, owing to the limited number of cases included in this study, there was a certain degree of bias, and future studies could expand the sample size for further analysis.

In terms of boundaries, there was a marked difference between the two tumor types. Some GSTs had blurred boundaries, whereas the majority of GSs had clear boundaries. This difference may be related to the malignant tendency of intermediate- or high-risk GSTs, which can invade and infiltrate surrounding tissues, resulting in blurred tumor boundaries and making the tumor indistinguishable from adjacent tissues. In terms of internal echogenicity, the internal echoes of GSTs are often heterogeneous. When the tumor was small, there were often small patchy areas of slightly hyperechoic or isoechoic regions within it. When the tumor diameter exceeded 5 cm, its interior may have exhibited cystic and solid components with strong echogenic foci, possibly due to necrosis, liquefaction, and calcium salt deposition within the enlarged tumor. This point can serve as one of the specific differentiating diagnostic features between the two tumor types. The internal echoes of the GSs were mostly homogeneous, which may be attributed to the faster growth rate of the GST than that of the GS, especially in intermediate- to high-risk GSTs, where intratumoral necrosis and calcification often occur, producing anechoic and strong echogenic regions. This observation aligned with the consensus on the internal echoes of GSTs and GSs reported in the literature (9, 10).

With respect to the preferential sites of tumor occurrence, GSTs were more commonly found in the lesser curvature of the gastric body and the antrum, with a smaller proportion occurring in the gastric fundus. In contrast, GSs were more frequently found in the antrum and gastric body, and only rarely in the gastric fundus. This difference may be related to the distribution patterns of Cajal and Schwann cells. Cajal cells are widely distributed throughout the gastric fundus, body, antrum, and pyloric canal, where they receive and transmit neural signals in the gastric fundus, body, and antrum and primarily participate in pacemaker activity in the pylorus. Therefore, due to this distribution pattern, GSTs are found in various parts of the stomach (4, 11). In contrast, Schwann cells are located in the Auerbach plexus between the circular and longitudinal muscle layers, with a richer distribution of nerves in the gastric body and antrum and relatively fewer nerves in the gastric fundus. This may explain the difference in the predominant sites of occurrence between GSTs and GSs.

GS is a relatively rare subtype of gastrointestinal mesenchymal tumors with an extremely low overall incidence rate, accounting for 0.2% to 0.5% of all gastric tumors and 5% to 10% of gastric mesenchymal tumors. It is the second most common gastric mesenchymal tumor next to GST, yet there is a significant disparity in the incidence rates between the two. Given the extremely low incidence of GS, only 16 cases were enrolled in the present study during the research period. To avoid statistical biases arising from unbalanced and small sample sizes, Firth’s penalized logistic regression was employed to address the unstable estimation of conventional logistic regression caused by complete separation. The results demonstrated that increased tumor diameter and unclear tumor boundary were significantly associated with GST, and unclear boundary remained an independent influencing factor in the multivariate model. To verify whether sample imbalance might compromise the stability of our conclusions, a sensitivity analysis was performed where the GS cohort was kept unchanged (n=16) and the GST cohort was randomly downsampled to 16 cases with 2000 repetitions. The analysis revealed that tumor diameter and boundary remained statistically significant in the vast majority of repetitions (the proportions of *p* < 0.05 were 0.913 and 0.795, respectively), indicating that the main conclusions had strong robustness to sample imbalance. In contrast, the low proportions of statistical significance for tumor morphology and internal echogenicity were more likely attributable to insufficient statistical power caused by reduced sample sizes after enforced sample balancing. To mitigate this limitation in future research, we propose conducting multi-center collaborations to expand the sample size for further validation and accumulate larger GS cohorts over an extended study period. Despite these limitations, our study provided valuable preliminary evidence for the differential diagnosis of GST and GS using gastric filling ultrasonography. The large sample size of the GST cohort (n=77) and pathologically confirmed diagnoses for all cases enhance the reliability of our primary findings. Further validation in larger, multi-center studies is warranted before the clinical implementation of the scoring system proposed in this study.

This study also analyzed the ultrasonographic features of GSTs of different risk levels. The results demonstrated significant differences among GSTs of varying risk levels in terms of average tumor diameter, internal echoes, morphology, boundaries, and blood flow distribution. Owing to their high degree of malignancy and rapid growth rate, intermediate- to high-risk GSTs have significantly larger tumor volumes than low-risk GSTs do. The internal echoes of intermediate- and high-risk GSTs were often heterogeneous, the boundaries were unclear, and morphologies were irregular and lobulated. Additionally, necrosis, liquefaction, and calcification were more commonly observed within these tumors, which is generally consistent with current consensus conclusions (12). The malignancy of GSTs is positively correlated with their size and mitotic count. GSTs smaller than 1 cm are often indolent with very low malignant activity, whereas those with larger diameters and higher mitotic counts are associated with increased malignancy. According to the National Institutes of Health (NIH) risk stratification, GSTs are classified into four categories: very low risk, low risk, intermediate risk, and high risk (7, 13). Among these, very low-risk GSTs rarely recur and are incidental findings during physical examinations in approximately 10%-35% of middle-aged and elderly individuals. These very low-risk GSTs have minimal clinical significance and do not require special attention. However, intermediate- to high-risk GSTs are highly malignant and prone to recurrence and metastasis even after complete surgical resection; thus, they should manage with targeted therapy or other treatments (8). According to the NCCN 2022 guidelines, the initial risk assessment of a GST with a diameter less than 2 cm can be accomplished via biopsy, and if no high-risk factors are identified, no intervention is needed; observation alone is sufficient. If the tumor is assessed as high-risk, surgical treatment should be performed promptly. Thus, the detection and monitoring of GSTs are equally important (8, 14). Therefore, the prognoses of GSTs vary significantly across different risk levels, making accurate risk assessment crucial for patients with newly diagnosed GSTs. To accurately distinguish GST with different risk stratification, ROC curve of average diameter in different degrees of malignancy was made and confirmed its cut-off value in our research. Then, cumulative score was acquired through the cut-off value of average diameter and other critical features such as internal echoes with fluid sonolucent area or strong echo spots, and the ROC curve of cumulative score between very low/low risk group and intermediate/high risk group was made again to determine the cut-off value, the result displayed when the score was greater than 3.5 points, GST risk stratification was intermediate or high risk, which provide a strong diagnostic basis for accurate risk assessment and their subsequent individualized treatment of newly diagnosed GST patients.

Previously, follow-up monitoring using CT or MRI had drawbacks, such as long examination times and high costs. The use of gastric filling ultrasonography effectively compensates for these deficiencies. In gastric filling ultrasonography, the origin of the lesion (submucosal or proper muscle layer), morphology (mostly oval or lobulated), boundary (clear or blurred, regular or irregular), presence or absence of ulceration and internal echoes (homogeneous or heterogeneous, with or without calcification or liquefaction) can be clearly visualized (9, 10, 15, 16). Furthermore, owing to the multiplane scanning capability of ultrasound, the volume of the lesion can be measured more accurately than can be measured via CT and MRI. Simultaneously, a comprehensive evaluation of the lesion’s blood flow, surrounding tissue invasion, and lymph node metastasis can be performed (9). Therefore, compared with CT or MRI, gastric filling ultrasonography is more portable, accurate, comprehensive, cost effective, repeatable, and meaningful for the monitoring of GSTs.

In addition, other studies have shown that there are also certain differences between the two tumor types in contrast-enhanced ultrasonography. Considerable research has been conducted on the use of contrast-enhanced harmonic endoscopic ultrasonography (CEH-EUS) in the diagnosis and management of GSTs, the arrival time for the GSTs was significantly faster than that of the surrounding normal gastric wall tissue, and most GSTs exhibited a ringlike hyperenhancement pattern on CEH-EUS images, with a peak intensity significantly greater than that of the surrounding normal gastric wall tissue. The sonographic appearance varies slightly depending on the risk level, with low-risk GSTs demonstrating weaker peak intensities than high-risk GSTs do (17, 18). Owing to the low incidence of GS, there is limited research on CEH-EUS for GS, with only a few cases reported describing peripheral ringlike hyperenhancement and homogeneous enhancement within the tumor (19). To date, no studies have been conducted on CEH-EUS for both GSTs and GSs. However, there is more research on differentiating between the two tumors using contrast-enhanced CT (CECT). According to CECT studies, there is no significant difference in enhancement between GSTs and GSs, but GSs exhibit moderate and homogeneous enhancement both before and after contrast agent administration, whereas GSTs demonstrate ringlike hyperenhancement with central necrosis (20). Owing to the limited number of patients who underwent gastrointestinal CEH-EUS at our hospital and the corresponding limited diagnostic experience, this study was unable to provide an in-depth analysis of GSTs and GSs using CEH-EUS. Instead, a comparative study was performed using conventional gastric filling ultrasonography, which constitutes a major limitation of this study. In future research, we plan to include more cases and conduct CEH-EUS studies on various submucosal gastric lesions, including GSTs and GSs, to provide a theoretical basis for their differential diagnosis.

## 5 Conclusions

Gastric filling ultrasonography can serve as a screening tool for initial diagnosis and follow-up monitoring and provide an important basis for the diagnosis and differential diagnosis of gastric submucosal lesions (including GSTs and GSs) and the prediction of GST risk stratification.

## Data Availability

The original contributions presented in the study are included in the article/supplementary material. Further inquiries can be directed to the corresponding author.
